# Progress and challenges to TB elimination in New South Wales, Australia

**DOI:** 10.5588/ijtldopen.24.0596

**Published:** 2025-06-13

**Authors:** E.J. Donnan, J. Pett, E. Ulbricht, P.D. Massey, V. Sintchenko, B.J. Marais

**Affiliations:** ^1^ Health Protection New South Wales, Health System Support Group, St Leonards, NSW, Australia;; ^2^ Sydney Infectious Diseases Institute (Sydney ID), The University of Sydney, Sydney, NSW, Australia;; ^3^ Respiratory and Sleep Medicine Department, Liverpool Hospital, South Western Sydney Local Health District, Liverpool, NSW, Australia;; ^4^ Hunter New England Population Health, Hunter New England Local Health District, Newcastle, NSW, Australia;; ^5^ College of Public Health, Medicine and Dentistry, James Cook University, Douglas, QLD, Australia;; ^6^ NSW Mycobacterium Reference Laboratory, New South Wales Health Pathology-Institute of Clinical Pathology and Medical Research, Westmead, NSW, Australia;; ^7^ Department of Infectious Diseases and Microbiology, The Children’s Hospital at Westmead, Westmead, NSW, Australia.

**Keywords:** tuberculosis, TBI, prevention/control programme, epidemiology, active case finding, migration, screening, transmission

## Abstract

In Australia, TB care and control is delivered by states and territories, with a National TB Advisory Committee to advise on national surveillance and strategy. For more than 30 years, New South Wales (NSW), Australia, has maintained TB incidence rates of <10/100,000 population, but progress toward TB elimination and ‘zero local TB transmission’ remains challenging. Reductions in the TB notification rate have plateaued in recent decades, mainly due to increased migration from high incidence countries. There is limited awareness of TB among the public, and a general perception of low risk, at least for Australian-born people and locally trained healthcare professionals. As in other low TB incidence settings, migrants and hard-to-reach populations are overrepresented in TB notifications. Progress in reducing TB among Australia’s Aboriginal and Torres Strait Islander people has been slow, hindered by embedded disadvantage, limited healthcare access and historical mistrust. Community engagement and patient advocacy for TB is minimal. Despite excellent progress over many decades, TB elimination remains out of reach in NSW due to ongoing migration from high-incidence settings and the reality of competing health priorities. Here, we critically assess progress towards TB elimination targets and identify opportunities to further improve TB control.

Australia is a low TB incidence country, with a WHO incidence estimate of 5.6 cases per 100,000 population in 2022.^1^ The state of New South Wales (NSW) accounts for 31.3% of the Australian population.^2^ Overseas-born people comprise 31.0% of the population, with high TB incidence countries including China, India, Philippines, Vietnam and Nepal among the top 10 countries of birth.^3^ Responsibility for health and communicable diseases sits with state and territory governments rather than the national government. The WHO created an action framework for low-incidence countries with eight priority actions for TB elimination.^4^ Australia reflected these WHO priorities in the ‘The Strategic Plan for Control of Tuberculosis in Australia, 2016–2020: Towards Disease Elimination’,^5-7^ whereas the 2021–2025 plan focuses more on ‘zero TB transmission’.^8^ A recent article presenting a renewed vision for TB elimination suggests that whilst all eight areas of the WHO framework are important, screening and appropriate treatment for TB and TB infection (TBI) in contacts and selected high-risk groups (Framework Area 4) are pivotal for elimination.^9^ We have therefore critically evaluated the WHO elimination action framework in the context of NSW. This has allowed us to consider how best to make progress in TB control as part of a series designed to provoke discussion on how to end the TB epidemic.^10^

## HISTORICAL PROGRESS IN TB CONTROL

**Figure 1.**
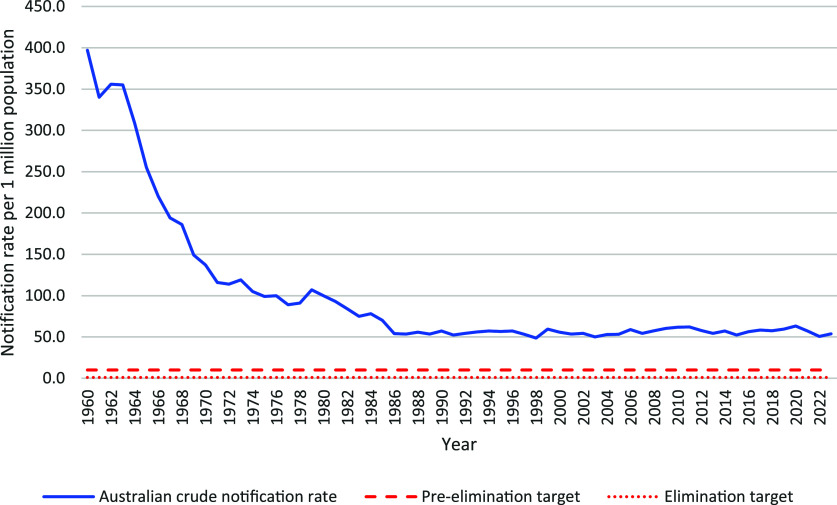
TB notification rate in Australia, 1960–2023. Source: Unpublished data provided by the Australian Department of Health and Aged Care, 17 September 2024.

**Figure 2.**
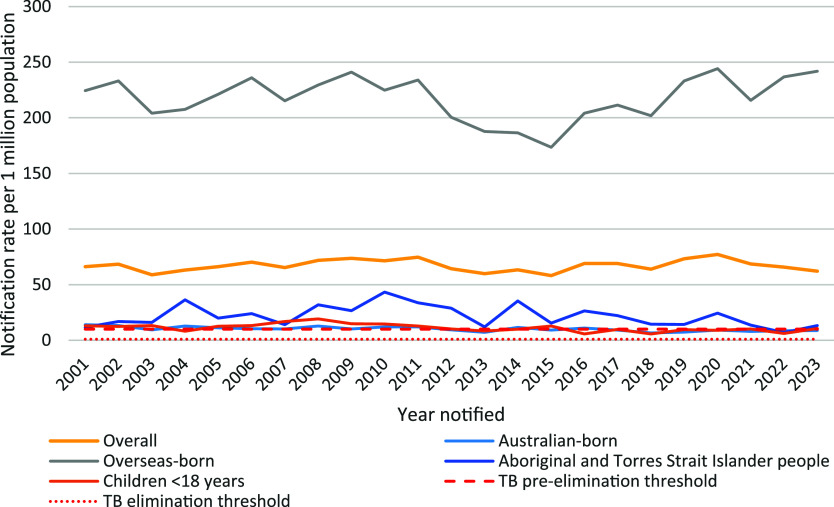
TB notification rate in key population groups in NSW, Australia, 2001–2023. Source: NSW Notifiable Conditions Information Management System, Australian Bureau of Statistics population estimates.

Australia’s National TB Campaign (1949–1975) was a highly successful partnership between the national, state and territory governments that reduced TB incidence by almost 75% from the 1960s to the early 1980s. However, TB notification rates have plateaued since the late 1980s in Australia (Figure 1)^8^ and NSW, slightly increasing between 2015–2020 and decreasing following the COVID-19 pandemic (Figure 2). Anecdotally, awareness of TB by public and healthcare professionals is low. The bacille Calmette-Guerin (BCG) vaccine was removed from the National Immunisation Program in 1980.^11^ Every year, there are examples of delayed diagnoses in both Australian-born and overseas-born people. We consider potential reasons for this in the following sections using the WHO elimination action framework.^4^

### Framework area 1: ensure political commitment, funding, and stewardship for planning and essential services

Australia does not have a national TB control programme; this is the responsibility of individual states and territories. The National Tuberculosis Advisory Committee (NTAC), a subcommittee of the Communicable Disease Network Australia, provides a mechanism for national oversight, coordination and strategic planning, but only in an advisory capacity.^8^ It is unclear at this point what role the new Australian Centres for Disease Control will have in relation to TB going forward.^12^ NSW Health is responsible for TB policies, guidelines, surveillance, monitoring and evaluation. All services related to the diagnosis and treatment of presumptive or confirmed TB (active or latent) must be provided free of charge to patients within the NSW public health system, regardless of their eligibility for Medicare benefits or visa status.^13^ Most clinical TB services are provided by hospital-based respiratory and infectious disease physicians and specialist nurses, with limited general practice (GP) involvement.^14^ Hospitals receive activity-based funding, which allocates resources based on the number and mix of patients treated. NSW Health requires health districts to have a specialist nurse to provide a coordination role, which may be combined with other roles, particularly in regional areas.^15^ Adequate community engagement and advocacy remains a challenge, which may be as a result of TB-related stigma in some communities.

### Framework area 2: address the most vulnerable and hard-to-reach groups

Hard-to-reach groups are classed as those whose socio-economic conditions or lifestyle make it difficult to recognise TB symptoms, access health services, and attend regular health care appointments.^4^ These groups may be characterised by shared cultural, medical or socio-economic factors.^5^ Routine TB surveillance data in NSW collects information such as country of birth, indigenous status and ethnicity (ethnicity was added in 2022). Other risk factors routinely collected include healthcare employment, immunosuppressive health condition or therapy, alcohol or other drug misuse, and history of residing in a correctional facility, shelter or being homeless. Groups that are considered high priority due to being overrepresented in TB notifications in NSW include Aboriginal and Torres Strait Islander Australians (Australia’s indigenous people) and Pasifika people (i.e., people of Samoan, Tongan, Fijian and Cook Islander heritage). Migrants born in countries with high TB incidence are covered in Area 3.

Aboriginal and Torres Strait Islander Australians are over-represented among people diagnosed with TB and among instances of local TB transmission as a consequence of embedded disadvantage, limited healthcare access in remote areas, and deep mistrust due to colonial occupation and dispossession.^16^ The need to reduce transmission and disease risk among Aboriginal and Torres Strait Islander people has been highlighted in numerous national strategic plans.^5,8,17^ Although progress has proven difficult, recent engagement with the National Aboriginal and Torres Strait Islander Health Protection subcommittee of the Australian Health Protection Committee represents an important advance.^18–21^ NSW Health, alongside other state and territory TB programmes, needs to continue to work to improve co-design with Aboriginal and Torres Strait Islander people to respond to TB inequity, the social determinants of health, and the historical, cultural, social, and structural processes relevant to TB care and control.^16^ An outbreak in regional NSW identified in 2000 still has occasional new cases.^22,23^ Of the 42 Australian-born people diagnosed with TB in NSW in 2023, four (10%) identified as an Aboriginal person. Although the number of people diagnosed with TB who identify as Aboriginal and Torres Strait Islander people varies year to year, the average TB notification rate over the past 10 years is double that of non-indigenous Australian-born people in NSW. Pasifika communities constitute approximately 1% of the population of NSW. Two large clusters representing local transmission of TB have predominantly involved Pasifika people in the past 10 years. One cluster affected migrants from various backgrounds between 2012–2018, then moved into the Pasifika community in 2019, with a rapid increase in linked cases between 2019 and 2022. There have been 26 disease episodes in the cluster, with 11 Pasifika people diagnosed, and no new diagnoses since 2022. A separate cluster appears to have started with an Asian-born temporary migrant in 2012 and then spread to the Samoan community, followed by 19 diagnoses from 2014–2021; 16 disease episodes were in members of an extended Samoan family and three in colleagues born in Australia or New Zealand.^24^ As ethnicity data (beyond Indigenous status) has only been collected since 2022, clusters in specific ethnic communities, particularly among those born in Australia or New Zealand, were previously more difficult to recognise.

### Framework area 3: address special needs of migrants and cross-border issues

TB overwhelmingly occurs in people born outside Australia, mostly due to reactivation following infection acquired prior to migration, or primary or re-infection during return visits.^25^ This is unsurprising given Australia’s strong connection and economic interdependence within the Indo-Pacific region and high mobility across the region. In 2023, 91.9% of people diagnosed with TB in NSW were born overseas, with the most common countries of birth being the Philippines (16.2%), India (15.0%), Nepal (13.6%), Vietnam (8.5%) and Australia (8.1%). The notification rate for overseas-born people was 20.3 per 100,000 population per year compared to 0.8 per 100,000 for Australian-born people. Almost half (46.2%) of overseas-born people diagnosed were permanent residents, 20.9% were overseas students, 6.3% were temporary visitors, 1.8% were refugees or people on a humanitarian visa, and 23.4% were on other visa types. A recent study emphasised that although disease risk is highest in the first two years post-migration, there is a persistent risk in later years.^26^ The median time from arrival in Australia to diagnosis in NSW was 6.5 years, ranging from 0 to 72 years; although this does not account for potential primary or re-infection during subsequent travel to their home country or other high TB incidence settings.^26^

**Figure 3.**
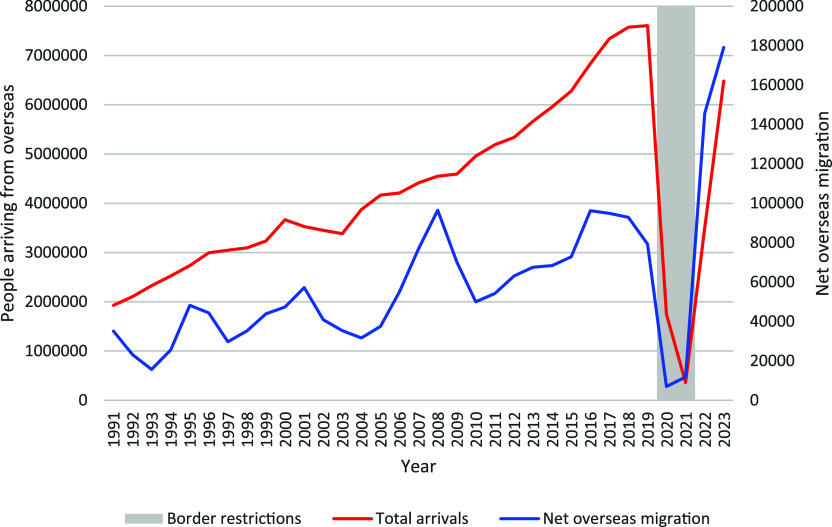
Total overseas arrivals and net overseas migration to NSW, Australia, 1991–2023. Source: Australian Bureau of Statistics Data Collection 3101.0 National, state and territory population and 3401.0 Total Arrivals and Departures, Australia.

**Figure 4.**
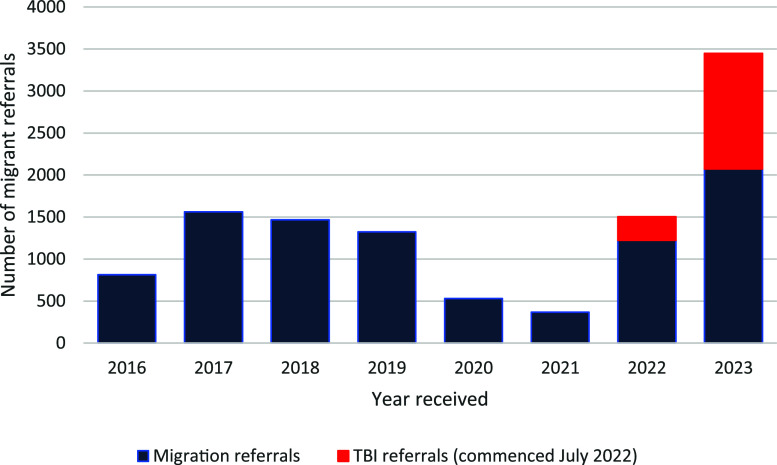
Referrals received by NSW Health for migrants requiring review following an immigration medical (TB) examination, 2016–2023. Source: Unpublished NSW TB Program, Health Protection NSW data.

People migrating to Australia for 6 months or more must be free from TB disease.^27–29^ People looking to migrate permanently, and some temporary visitors, are required to undergo medical examinations. Those migrating from countries with a TB incidence ≥40 per 100,000 and ≥11 years of age are required to have a chest X-ray (CXR). Children aged 2–10 years are required to have a tuberculin skin test (TST) or interferon-gamma release assay (IGRA) test for TBI, followed by a CXR in those where TBI is detected. Since July 2022, migrants from countries with a WHO-estimated TB incidence greater or equal to 40 per 100,000 population per year who intend to work, study or train to be a healthcare worker or work within healthcare, aged care, disability or childcare facility in Australia require a TBI test.^30^ Effective migration screening reduces the number of people with TB disease entering Australia, but may increase TB stigma. There are anecdotal reports of patients highly concerned that their TB diagnosis may compromise their visa status. The concerns are unfounded but may reduce willingness to present with clinical signs and symptoms of TB and may lead to people seeking treatment overseas. The number of TB diagnoses in NSW is highly susceptible to changes in migration and migration policy. TB notifications increased in the years 2015–2020, following increases in migration, with a growing proportion from high TB incidence countries, which was followed by strict travel restrictions during the COVID-19 pandemic (Figure 3). Although there were fewer new arrivals during this time, many students and visitors from high TB incidence countries remained in Australia under stressful circumstances. This, in combination with increased vigilance and focus on investigating respiratory illness, may explain why border closures did not result in an immediate decline in TB incidence. TB notifications decreased in 2021–2022, likely due to the impact of border closures on migration and meticulous pre-migration screening due to the COVID-19 pandemic (Figure 4).^31^ In November 2022, there was a streamlining of requirements for temporary visa applicants and most temporary visa applicants were no longer required to undertake a medical examination or CXR.^32^ This lasted until November 2023 and may have reduced TB notifications during this period. However, net migration and referrals from pre-migration screening significantly increased in 2023; 106 migrants were diagnosed with TB disease in NSW following pre-migration screening or follow-up. This constituted 22.2% of TB diagnoses in overseas-born people in NSW in 2023. There can be major diagnostic delays for migrants who are not eligible for subsidised healthcare in Australia due to perceived costs. Although TB care is free in NSW public hospitals, costs may be incurred if the initial investigations and diagnosis are undertaken in a private healthcare facility or service, such as general practice. Diagnostic delay due to real or perceived costs, or unfounded fear that it may impact their visa status, worsen patient outcomes and increase local transmission risk.

### Framework area 4: undertake screening and appropriate treatment for TB and TB infection in contacts and selected high-risk groups

**Figure 5.**
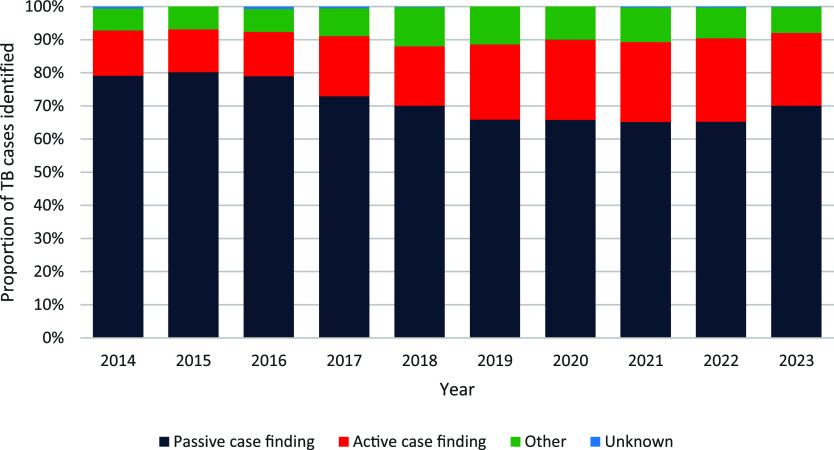
Proportional contribution of different tuberculosis case detection methods in NSW, Australia, 2014–2023. Source: NSW Notifiable Conditions Information Management System.

Increasing active case-finding and the proportion of people diagnosed with TB through screening is a hallmark of a well-functioning TB control programme, particularly if people are identified with subclinical TB before they become infectious.^33^ TB screening is undertaken in selected high-risk groups in NSW, and migrant screening is described above. TBI screening is routinely undertaken in: close TB contacts; healthcare workers or people undertaking clinical placements in public health facilities; people with HIV; people commencing treatment with specific biological agents; people preparing for organ transplantation; and renal patients. In 2023, passive case-finding (i.e., people presenting with clinical symptoms) was the most frequent reason for diagnosis (70.1%) in NSW, whereas active case-finding accounted for only 21.9% of people diagnosed. The proportion of people notified with TB who were diagnosed through passive case finding has decreased by 8% over the past 10 years, whereas the proportion found through active case finding increased by 9% (Figure 5). Increasing active case-finding should reduce the overall public health risk due to TB by identifying either subclinical or early TB disease before a person becomes highly infectious. In addition to diagnosing subclinical disease, the time from onset of symptoms to initiation of effective TB treatment should also be as short as possible to limit disease progress and transmission. The performance of the health system can be monitored by the time from first healthcare contact for TB symptoms to treatment. In NSW in 2023, the median time from first healthcare contact to treatment initiation was 24 days for Australian-born people and 27 days for those born overseas.

Contact screening for TB is a public health requirement, and NSW guidelines are in place to support this.^13,34^ Aggregated contact screening outcomes are routinely collected. In 2023, 2,655 contacts were identified from 272 laboratory-confirmed pulmonary TB cases, of which 62.9% completed contact screening. This elicited 17 people with TB disease and 274 people with TBI. Contact screening completeness in NSW ranges from 55–65%, with large variation in the number of contacts identified across the years.^31^ Further investigation is being undertaken into factors that may affect contact screening completeness such as clinic and geographic area, demographic characteristics and contact methods used and this may allow for specific, focused strategies to improve screening completion. However, routine whole genome sequencing reveals only a small number of secondary cases not detected during contact screening and these are actively monitored. Establishing flexible mechanisms to provide public health surge capacity was an important lesson from the COVID-19 pandemic, and is also valuable when large TB contact investigations are necessary.^35^

### Framework area 5: optimise the prevention and care of drug-resistant TB

Mycobacterium Reference Laboratories (MRLs) fulfil an important TB control function.^36,37^ The NSW Health Pathology Institute of Clinical Pathology and Medical Research provides mycobacterial culture, speciation, phenotypic drug susceptibility testing (DST), rapid molecular methods and routine whole-genome sequencing (WGS) for *Mycobacterium tuberculosis*. In 2023, of the 520 people diagnosed with TB, 78.8% were laboratory-confirmed; 68.7% by *M. tuberculosis* culture, 10.2% by detection of *M. tuberculosis* by nucleic acid amplification test (NAAT) only, and 21.2% were clinically diagnosed. Of the culture-positive cases, 98.3% had DST results; 86.9% were fully susceptible, 9.7% were resistant to one or more first-line TB drugs, and 3.4% (12 cases) were classified as multidrug-resistant TB (MDR-TB). Most rifampicin-resistant TB (RR-TB) or MDR-TB cases were identified early by rapid molecular tests (mostly Xpert^®^ MTB/RIF or Ultra; Cepheid, Sunnyvale, CA, USA), although in some sputum smear-negative cases, it was only detected once cultured.

Access to medications as per the Australian recommendations for the Management of Drug-Resistant Tuberculosis is generally good, with shorter, all oral MDR/RR-TB regimens available where indicated.^38^ Most second-line agents are not on the Australian Register of Therapeutic Goods, and access must be requested through the Special Access Scheme.^39^ Occasional delays in drug procurement exist where bridging regimens may be required. BPaL/M was used in 10 of the 12 MDR/RR-TB cases in NSW in 2023, indicating a strong uptake of the shorter, all-oral regimens. MDR-TB treatment outcomes in NSW have been excellent: between 2013 and 2022, 98.6% of MDR/RR-TB cases successfully completed treatment.

### Framework area 6: Ensure continued surveillance, programme monitoring, and evaluation and case-based data management

TB is a notifiable condition under the NSW *Public Health Act* 2010; doctors are required to notify all persons where they reasonably suspect TB and laboratories are required to notify positive culture and NAAT results. Enhanced surveillance data, including demographic and clinical information, risk factors, and additional laboratory information (*M. tuberculosis* DST and WGS), are collected in NCIMS, with additional cluster information in a REDCap database. An epidemiologist undertakes routine surveillance and reporting with quarterly and annual key performance indicators and reports.^40^ Further data on TBI diagnoses and management would be useful to monitor progress. There is an absence of good benefit-risk data for individuals of all ages, given increased adverse drug reactions associated with TB preventive treatment in older people and uncertainty about the durability of the effect in settings with high rates of return travel to countries with a high burden of TB and potential for re-infection. A limitation is the disparate systems, including public and private hospitals, community health, primary care and population health surveillance systems. Although almost all TB patients are managed in public hospitals, districts use different medical record systems that preclude the extraction of state-wide hospital data. Detailed data on TBI testing and treatment, and contact screening are not available. A statewide electronic medical record system is planned.

### Framework area 7: invest in research and new tools

A committed research community is maintained by the Australian National Health and Medical Research Council-funded Centre for Research Excellence in TB Control (TB-CRE),^41^ and the WHO Collaborating Centre for TB hosted by the University of Sydney Infectious Diseases Institute (Sydney ID).^42^ The Australasian Clinical Tuberculosis Network (ACTNet) is a collaborative group that supports and links TB practitioners in Australia and New Zealand, but individual researchers remain responsible for developing and supporting research activities.^43^ The NSW Health Centre for Infectious Diseases and Microbiology - Public Health has undertaken extensive research into the molecular epidemiology of *M. tuberculosis,* and NSW was one of the pioneers in implementing routine genomic surveillance of communicable diseases.^44–46^ WGS was available for 98.6% of culture-confirmed TB cases in NSW in 2023; 10.3% were in a cluster with another isolate/s in NSW. There were 30 different clusters with a new case added in 2023; 46.6% of clusters involved two cases, 40.0% three or four cases, and 13.3% contained five or more cases. There is further scope to maximise the utility of WGS for cluster detection, monitoring and evaluation purposes; however, implementation barriers at the national level need to be overcome to realise their full potential.^47^

### Framework area 8: support global TB prevention, care and control

State-based services do not directly support global TB elimination efforts, but many clinicians are keen to contribute to global prevention and control efforts. The TB-CRE and other Australian research groups contribute, particularly in South East Asia and the Western Pacific regions.^48^ The Australian Government supports regional TB programmes and control efforts through the Department of Foreign Affairs and Trade, such as $AUD 17 million given to the Global Alliance for Tuberculosis in 2024.^49^ There are multiple TB-related projects led by Australian researchers in countries such as Vietnam, Indonesia, Malaysia, East Timor, Papua New Guinea and Kiribati.^50–52^

## CONCLUSION

For almost 40 years, TB notification rates in NSW have plateaued at 5–7 cases per 100,000 population per year, despite numerous national strategic plans and calls for TB elimination.^5,8^ The Strategic Plan for Control of Tuberculosis in Australia, 2016–2020, set out Australia’s vision and milestones to achieve the End TB targets for low-incidence countries alongside an ambitious work plan.^5^ Some actions from the 2016-2020 strategic plan were completed, but many were still in progress and informed key actions in the 2021–2025 strategic plan.^8^

Overall, NSW has made good progress against the WHO framework actions by including universal contact investigation, prevention and treatment of TB in migrants, optimising care for drug-resistant TB, and establishing improved surveillance and programme monitoring. However, similar to the European experience,^53^ there has been stagnation in some areas.^4^ Based on a TB incidence of 1–9 cases per 100,000 population, NSW is at stage 4 of Migliori et al.’s proposed five-stage framework for near-term progress towards TB pre-elimination.^9^ Most of the key priorities and actions required at this stage are embedded in policy in NSW, though some implementation challenges remain (Table 1). Important opportunities to enhance TB elimination efforts include minimising TB disease progression and transmission in NSW through better screening, increased TB preventive treatment use, early disease detection, concerted efforts to terminate local transmission clusters, and performing relevant consumer-led operational research and health promotion activities in affected communities. Australia’s focus on ‘zero local TB transmission’ provides an opportunity to redefine TB elimination in a way that embraces innovation and new technology, with continued investment in TB services to prevent imported disease from spreading. Knowing that the Australian population is safe from the risk of locally transmitted TB disease, allows us to create a welcoming atmosphere for migrants.

**Table.** NSW policy and implementation progress against TB pre-elimination priority actions and key interventions.^9^

	Item	Policy	Strategy/implementation
Priority actions	Eliminate within-country transmission	Yes	WGS monitoring in place identifying some transmission and with some ongoing outbreaks
	Treat *M. tuberculosis* infection in all risk groups	Yes	Further upscaling of TPT required
	Prevent and treat TB in migrants	Yes	Improved pre-migration screening and further upscaling of TPT in key risk groups required
Key interventions	Universal contact investigation of people with infectious TB	Yes	NSW achieved 63% contact screening completeness in 2023 with 55% TPT uptake in those found to have TBI (89% of child contacts <5 years screened with 100% uptake of TPT in those found to have TBI)
	Treat TB infection in other high-risk groups (e.g. people with poorly controlled diabetes or using tumour necrosis factor blockers)	Yes	Generally good infection testing and TPT uptake pre-immunosuppression, e.g. for people requiring transplants or TNF blockers treatment for autoimmune conditions
			Unclear for more common conditions such as silicosis and diabetes
	Test non-native-born people for infection	Partial	Children aged 2–10 years, and people anticipating working or studying in healthcare, disability, aged care or childcare tested pre-migration
			NSW Health workers, students and volunteers born or who have lived or travelled to countries with TB incidence ≥40,000/100,000 for ≥3 months tested pre-employment
			Generally low TPT uptake and uncertain risk-benefit in certain groups, such as older people

WGS = whole-genome sequencing; TPT = TB prevention therapy; TBI = TB infection; TNF = tumour necrosis factor; NSW = New South Wales.
